# Death of a Parent and the Risk of Ischemic Heart Disease and Stroke in Denmark and Sweden

**DOI:** 10.1001/jamanetworkopen.2022.18178

**Published:** 2022-06-22

**Authors:** Hua Chen, Jiong Li, Dang Wei, Mikael Rostila, Imre Janszky, Yvonne Forsell, Tomas Hemmingsson, Krisztina D. László

**Affiliations:** 1 Department of Global Public Health, Karolinska Institutet, Stockholm, Sweden; 2 Department of Clinical Medicine–Department of Clinical Epidemiology, Aarhus University, Aarhus, Denmark; 3 Department of Public Health Sciences, Stockholm University, Stockholm, Sweden; 4 Centre for Health Equity Studies, Stockholm University/Karolinska Institutet, Stockholm, Sweden; 5 Department of Public Health and Nursing, Faculty of Medicine, Norwegian University of Science and Technology, Trondheim, Norway; 6 Centre for Epidemiology and Community Medicine, Stockholm County Council, Stockholm, Sweden; 7 Institute of Environmental Medicine, Karolinska Institutet, Stockholm, Sweden

## Abstract

**Importance:**

Increasing evidence suggests that parental death is associated with unhealthy behaviors and mental ill-health. Knowledge regarding the link between parental death and the risk of ischemic heart disease (IHD) and stroke remains limited.

**Objectives:**

To investigate whether parental death is associated with an increased risk of IHD and stroke and whether these associations differ by the characteristics of the loss.

**Design, Setting, and Participants:**

This population-based cohort study, involving linkages between several nationwide registers, included 3 766 918 individuals born between 1973 and 1998 in Denmark and between 1973 and 1996 in Sweden. Participants were followed up until 2016 in Denmark and 2014 in Sweden. Data were analyzed from December 2019 to May 2021.

**Exposures:**

Death of a parent.

**Main Outcomes and Measures:**

Diagnosis with or death due to IHD or stroke. Poisson regression was used to analyze the associations between parental death and IHD and stroke risk.

**Results:**

Altogether, 48.8% of the participants were women, and 42.7% were from Denmark. A total of 523 496 individuals lost a parent during the study period (median age at loss, 25 years; IQR, 17-32 years). Parental death was associated with a 41% increased risk of IHD (incidence rate ratio [IRR], 1.41; 95% CI, 1.33-1.51) and a 30% increased risk of stroke [IRR, 1.30; 95% CI, 1.21-1.38). The associations were observed not only if the parent died because of cardiovascular or other natural causes but also in cases of unnatural deaths. The associations were stronger when both parents had died (IHD: IRR, 1.87; 95% CI, 1.59-2.21; stroke: IRR, 1.64; 95% CI, 1.35-1.98) than when 1 parent had died (IHD: IRR, 1.37; 95% CI, 1.28-1.47; stroke: IRR, 1.27; 95% CI, 1.19-1.36) but did not differ substantially by the offspring’s age at loss or the deceased parents’ sex. The risk of acute myocardial infarction was highest in the first 3 months after loss.

**Conclusions and Relevance:**

In this cohort study, parental death in the first decades of life was associated with an increased risk of IHD and stroke. The associations were observed not only in cases of parental cardiovascular and other natural deaths but also in cases of unnatural deaths. Family members and health professionals may need to pay attention to the cardiovascular disease risk among parentally bereaved individuals.

## Key Points

**Question:**

Is parental death associated with increased risks of ischemic heart disease and stroke, and do these associations differ by the characteristics of the loss?

**Findings:**

In this cohort study involving 3.7 million individuals in Sweden and Denmark, parental death was associated with a 41% increased risk of ischemic heart disease and a 30% increased risk of stroke. The associations were observed not only if the parent died because of cardiovascular or other natural causes but also in cases of unnatural death.

**Meaning:**

These findings suggest that affected individuals, their family members, and health professionals may need to pay attention to the increased cardiovascular risk among the parentally bereaved.

## Teaser

This cohort study examines whether the death of a parent is associated with increased risks of ischemic heart disease and stroke among individuals in Denmark and Sweden.

## Introduction

Compelling evidence suggests that death of a spouse in middle and old age is associated with an increased risk of cardiovascular mortality.^1^ A few studies have also reported increased risks of ischemic heart diseases (IHDs), stroke, and atrial fibrillation after the loss of a spouse,^2,3^ sibling,^4,5^ or child.^6^ However, knowledge regarding the association between parental death and incident cardiovascular diseases (CVDs) is limited.

Parental death during childhood, a life event that affects 3% to 4% of children in the Nordic countries, is one of the most traumatic events that a child can experience.^7^ Parental death in childhood may result in disruptions in daily routines, poor psychological functioning, low social support, financial insecurity, and changes in the living environment in childhood, as well as attachment difficulties with a partner, lower educational attainment, and unemployment in adulthood.^8,9,10^ Several studies suggest that losing a parent in childhood or in early adulthood is associated with elevated stress reactivity^11^ and increased risks of psychological and behavioral disorders,^12^ hypertension,^11^ and metabolic syndrome,^13^ which in turn may increase the risk of CVD in adulthood.^10^ Studies investigating potential health consequences of parental death have focused generally on childhood exposure, probably because losing a parent in adulthood is regarded as a universal experience. Nevertheless, parental death in adulthood may also have a strong emotional impact and implications for cardiovascular health.

To our knowledge, only 1 study has investigated whether parental death is associated with an increased risk of incident IHD or stroke. Chen et al^10^ found in a cohort of Swedish men born between 1949 and 1951 that study participants who lost a parent in childhood had an increased risk of IHD, but not of stroke, up to the age of 57 to 59 years. To what extent findings from that study may be generalized to cohorts born in later periods or to women is unclear. Growing up during later historical periods and thus during more recent phases of development of the Nordic welfare state involves access to a wider range of family welfare policies that may buffer the negative effects of parental death during childhood.^14^ Women may be more strongly affected psychologically by losses of close emotional relationships^15^ and may be more sensitive physiologically to stress than men^16^; thus, the association between parental death and CVD may be stronger in women than that reported by Chen et al.^10^

In this study based on a large cohort born during the 1970s through the 1990s in 2 Nordic countries, we investigated whether parental death up to the offspring age of 43 years was associated with increased risks of IHD and stroke and whether these associations differed by characteristics of the loss.

## Methods

### Study Population and Design

This cohort study’s population consisted of persons recorded in the Danish Medical Birth Register from 1973 through 1998 and in the Swedish Medical Birth Register from 1973 through 1996 (n = 4 089 306). As the incidences of IHD and stroke are very low in childhood, we studied only individuals who reached the age of 18 years by the end of our follow-up periods (2016 in Denmark and 2014 in Sweden) to allow for a fair chance to develop the outcomes. We linked the birth registers to several nationwide, population-based registers to obtain information on demographic, socioeconomic, and health-related factors for the cohort and their family members (eTable 1 in the Supplement). Linkage was possible through the unique personal number assigned to all Danish and Swedish residents. We used the Danish Medical Birth Register, the Danish Civil Registration System, the Swedish Medical Birth Register, and the Swedish Multi-Generation Register to identify study participants’ family members (parents, grandparents, and aunts and uncles). We excluded participants for whom we did not have information on the father (n = 322 388). The study population thus consisted of 3 766 918 participants (1 608 292 from the Danish Medical Birth Register and 2 158 626 from the Swedish Medical Birth Register).

The study was approved by the Danish Data Protection Agency (reference numbers 2008-41-2680 and 2013-41-2569) and the Regional Ethical Review Board in Stockholm (reference number 2016/288-31/1). The boards did not request written consent for analyses involving anonymized, register-based data. The study was reported in accordance with the Strengthening the Reporting of Observational Studies in Epidemiology (STROBE) reporting guideline for cohort studies.

### Measures

#### Exposure

We defined exposure as the death of a parent during the study period. We obtained information on parents’ date and cause of death from the Danish Civil Registration System and from the Swedish Cause of Death Register. We classified exposure according to (1) the sex of the deceased parent, (2) the parent’s cause of death (CVD, other natural causes, or unnatural causes) (eTable 2 in the Supplement), and (3) the participant’s age at parental loss (≤5, 6-12, 13-18, 19-25, 26-30, or >30 years). Unnatural deaths included deaths due to violence, suicide, accident, complication of medical and surgical care, and other sudden deaths due to unknown causes. In case participants lost both parents during the follow-up, we considered the first loss in the analyses. Because mothers are generally the primary attachment figures and the main caregivers, we classified the loss as that of the mother if both parents died on the same day. We also classified exposure according to the number of deceased parents during follow-up.

#### Outcome

We obtained information on IHD and stroke from the National Hospital Register and the Civil Registration System in Denmark and from the Patient Register and the Cause of Death Register in Sweden. We searched the primary diagnoses and the underlying causes of death for the *International Classification of Diseases* codes presented in eTable 2 in the Supplement. We followed participants from birth until the first occurrence of the studied outcomes, death due to other causes, emigration, or end of the study periods (December 31, 2016, in Denmark and December 31, 2014, in Sweden), whichever came first. The source of information on emigration is presented in eTable 1 in the Supplement.

#### Covariates

We obtained information on demographic, socioeconomic, and health-related factors on the cohort and their family members as described in eTable 1 and eAppendix 1 in the Supplement. The* International Classification of Diseases* codes concerning medical conditions are presented in eTable 2 in the Supplement and the categorization of each covariate in Table 1.

**Table 1.** Characteristics of the Study Population According to Exposure to Death of a Parent

Variable	Participants by exposure status, No. (%)	
	Unexposed (n = 3 243 422)	Exposed (n = 523 496)
Country		
Denmark	1 345 131 (41.5)	263 161 (50.3)
Sweden	1 898 291 (58.5)	260 335 (49.7)
Sex		
Men	1 660 367 (51.2)	268 477 (51.3)
Women	1 582 964 (48.8)	255 013 (48.7)
Missing	91 (<0.1)	6 (<0.1)
Gestational age for the study participants, wk		
<32	21 061 (0.6)	3676 (0.7)
32-36	138 777 (4.3)	23 005 (4.4)
>36	2 798 948 (86.3)	372 133 (71.1)
Missing	284 636 (8.8)	124 682 (23.8)
Parents’ country of birth^a^		
Denmark or Sweden	2 839 670 (87.6)	448 016 (85.6)
Other countries	403 752 (12.4)	75 480 (14.4)
Parents’ highest educational level, y^b^		
0-9	248 820 (7.7)	95 071 (18.2)
10-14	1 959 133 (60.4)	306 659 (58.6)
≥15	1 023 299 (31.5)	120 121 (22.9)
Missing	12 170 (0.4)	1645 (0.3)
Maternal income at study participant’s birth^c^		
Low tertile	1 027 392 (31.7)	172 753 (33.0)
Middle tertile	1 072 986 (33.1)	147 766 (28.2)
High tertile	1 049 672 (32.4)	151 907 (29.0)
Missing	93 372 (2.9)	51 070 (9.8)
Maternal age at study participant’s birth, y		
<20	105 182 (3.2)	19 705 (3.8)
20-24	789 552 (24.3)	114 193 (21.8)
25-29	1 267 919 (39.1)	175 214 (33.5)
30-34	787 029 (24.3)	135 379 (25.9)
>34	293 740 (9.1)	79 005 (15.1)
Maternal smoking in early pregnancy		
No	1 196 743 (36.9)	66 273 (12.7)
Yes	410 010 (12.6)	46 462 (8.2)
Missing	1 636 669 (50.5)	410 761 (78.5)
Maternal BMI in early pregnancy		
<30	744 897 (23.0)	55 521 (10.6)
≥30	37 207 (1.1)	3368 (0.6)
Missing	2 461 318 (75.9)	464 607 (88.8)
Maternal hypertensive disorder before or during pregnancy		
No	2 941 762 (90.7)	415 415 (79.4)
Yes	116 449 (3.6)	15 078 (2.9)
Missing	185 211 (5.7)	93 003 (17.8)
Maternal diabetes before or during pregnancy		
No	3 045 272 (93.9)	427 894 (81.7)
Yes	12 939 (0.4)	2599 (0.5)
Missing	185 211 (5.7)	93 003 (17.8)
Parents’ CVD before study participant’s birth		
No	2 976 319 (91.8)	414 329 (79.1)
Yes	81 892 (2.5)	16 164 (3.1)
Missing	185 211 (5.7)	93 003 (17.8)
Parents’ psychiatric disorders before study participant’s birth		
No	3 140 353 (96.8)	494 016 (94.4)
Yes	103 069 (3.2)	29 480 (5.6)
Family history of CVD before study participant’s birth		
No	1 220 573 (37.6)	124 748 (23.8)
Yes	1 219 932 (37.6)	141 275 (27.0)
Missing	802 917 (24.8)	257 473 (49.2)
Family history of psychiatric disorders before study participant’s birth		
No	1 820 201 (56.1)	182 331 (34.8)
Yes	535 130 (16.5)	64 344 (12.3)
Missing	888 091 (27.4)	276 821 (52.9)

Abbreviations: BMI, body-mass index (calculated as weight in kilograms divided by height in meters squared); CVD, cardiovascular disease.

^a^ Classified according to whether both parents were from the studied countries (Denmark or Sweden).

^b^ Defined as the highest education of the 2 parents; if information on education was missing for 1 of the parents, we used information only from the other parent.

^c^ Classified based on the tertile distribution of each calendar year.

### Statistical Analyses

Data were analyzed from December 2019 to May 2021. We performed Poisson regression models to analyze the associations between parental death and the risks of IHD and stroke as described in more detail in eAppendix 2 in the Supplement. We ran analyses with any loss and with exposure categorized according to the sex of the deceased parent, parent’s cause of death, bereaved participant’s age at loss, and number of deceased parents during follow-up; the unexposed group was regarded as the reference. We ran models adjusted for time since birth and calendar year and models additionally adjusted for country, maternal age at participant’s birth, parents’ country of origin, highest education, and history of psychiatric disorders before participant’s birth.

We investigated the importance of the time since loss on the risk of IHD, stroke, and acute myocardial infarction (AMI) by repeating our main models after splitting time since loss as less than 3 months, 3 months to 1 year, 1 to 5 years, 5 to 10 years, and more than 10 years. Given that information on several potential confounders was not available for the whole cohort (eTable 1 in the Supplement), we adjusted for these measures in sensitivity analyses restricted to those with data on these variables. We ran analyses stratified by sex, country, and highest parental education. We ran analyses (1) with AMI and ischemic stroke, subarachnoid hemorrhage, and intracerebral hemorrhage separately; (2) after excluding individuals with 2 parents dying on the same day; and (3) including only the first child of each family so as to investigate the importance of familial clustering. We used SAS, version 9.4 (SAS Institute Inc) for the analyses.

## Results

Of the 3 766 918 study participants (48.8% women and 51.2% men; 42.7% from Denmark and 57.3% from Sweden), 523 496 (13.9%) experienced parental death during the study period (median age at loss, 25 years [IQR, 17-32 years). Compared with their unexposed counterparts, participants who had experienced parental death were more likely to have parents born in countries other than Denmark or Sweden (14.4% vs 12.4%), with lower educational attainment (≤9 years: 18.2% vs 7.7%) and with psychiatric disorders (5.6% vs 3.2%), and were more likely be born to older mothers (maternal age at birth >34 years: 15.1% vs 9.1%) (Table 1).

### Main Analyses

A total of 5915 (5.5 per 100 000 person-years) study participants had IHD, and 8154 (7.5 per 100 000 person-years) had a stroke during the mean (SD) 28.8 (8.6) years of follow-up. The median age at diagnosis was 32 years (IQR, 25-36 years) for IHD and 26 years (IQR, 19-33 years) for stroke. Participants who experienced parental death had a 41% higher risk of IHD and a 30% higher risk of stroke than their unexposed counterparts (adjusted incidence rate ratios [IRRs], 1.41 [95% CI, 1.33-1.51] and 1.30 [95% CI, 1.21-1.38], respectively) (Table 2). The IRRs were highest when the parent died because of CVD (for participants with IHD, 2.27 [95% CI, 2.05-2.52]; for participants with stroke, 1.50 [95% CI, 1.32-1.69]) but were increased also after losses due to unnatural causes (for participants with IHD, 1.26 [95% CI, 1.08-1.46]; for participants with stroke, 1.38 [95% CI, 1.20-1.58]). The associations did not differ by the deceased parent’s sex. Parental death both in childhood and in adulthood was associated with increased risks of IHD and stroke. The point estimates corresponding to parental death in the first 5 years of life and in adolescence were slightly higher than those corresponding to losses in early adulthood (19-43 years). However, the CIs for these periods largely overlapped (Table 2). There were no substantial differences in the associations when we performed more in-depth analyses according to the sex of the deceased parent (eTable 3 in the Supplement) or the child’s age at loss (eTable 4 in the Supplement). Risks for IHD and stroke were higher in cases of loss of both parents (IHD: IRR, 1.87; 95% CI, 1.59-2.21; stroke: IRR, 1.64; 95% CI, 1.35-1.98) than loss of 1 parent (IHD: IRR, 1.37; 95% CI, 1.28-1.47; stroke: IRR, 1.27; 95% CI, 1.19-1.36) (Table 2).

**Table 2.** Adjusted IRRs and 95% CIs for Ischemic Heart Disease and Stroke According to Parental Death

Exposure	Ischemic heart disease				Stroke			
	No.	Events/person-years	IRR (95% CI)		No.	Events/person-years	IRR (95% CI)	
			Model 1^a^	Model 2^b^			Model 1^a^	Model 2^b^
Unexposed	3 286 040	4609/103 286 808	1 [Reference]	1 [Reference]	3 286 224	7002/103 256 190	1 [Reference]	1 [Reference]
All deaths	480 878	1306/5 207 248	1.65 (1.55-1.76)	1.41 (1.33-1.51)	480 694	1152/5 205 661	1.39 (1.30-1.48)	1.30 (1.21-1.38)
Cause of death^c^^,^^d^								
Death due to CVD	98 958	422/1 062 167	2.44 (2.21-2.70)	2.27 (2.05-2.52)	98 912	277/1 062 353	1.55 (1.37-1.75)	1.50 (1.32-1.69)
Other natural death	290 740	682/2 928 225	1.40 (1.29-1.52)	1.18 (1.09-1.28)	290 590	654/2 926 965	1.30 (1.20-1.42)	1.21 (1.11-1.31)
Unnatural death	74 315	183/1 186 174	1.50 (1.29-1.74)	1.26 (1.08-1.46)	74 307	214/1 185 694	1.49 (1.30-1.71)	1.38 (1.20-1.58)
Participant’s age at loss, y^d^								
0-5	27 942	85/733 965	2.01 (1.62-2.49)	1.57 (1.25-1.96)	27 942	85/733 822	1.46 (1.18-1.81)	1.36 (1.10-1.69)
6-12	54 214	142/1 103 513	1.67 (1.41-1.97)	1.35 (1.14-1.60)	54 208	161/1 103 193	1.46 (1.25-1.71)	1.34 (1.14-1.57)
13-18	76 011	219/1 082 234	1.86 (1.62-2.13)	1.58 (1.38-1.81)	75 988	219/1 081 785	1.53 (1.34-1.75)	1.42 (1.24-1.63)
19-25	114 725	301/1 176 799	1.56 (1.38-1.75)	1.34 (1.19-1.51)	114 671	281/1 176 231	1.34 (1.18-1.51)	1.25 (1.10-1.41)
26-30	82 495	275/620 141	1.78 (1.57-2.01)	1.54 (1.36-1.75)	82 439	189/620 065	1.28 (1.10-1.48)	1.20 (1.03-1.39)
>30	125 491	284/490 596	1.42 (1.25-1.61)	1.26 (1.11-1.43)	125 446	217/490 565	1.34 (1.16-1.54)	1.27 (1.10-1.46)
Sex of the deceased parent^d^								
Female (mother)	153 544	402/1 628 649	1.58 (1.42-1.75)	1.35 (1.21-1.50)	153 496	381/1 628 122	1.44 (1.29-1.59)	1.34 (1.20-1.49)
Male (father)	327 334	904/3 578 599	1.69 (1.57-1.82)	1.45 (1.34-1.56)	327 198	771/3 577 540	1.37 (1.27-1.48)	1.28 (1.18-1.38)
No. of deceased parents during the study period^d^								
One parent	441 128	1142/4 944 847	1.58 (1.48-1.69)	1.37 (1.28-1.47)	440 953	1038/4 942 951	1.35 (1.26-1.45)	1.27 (1.19-1.36)
Both parents	39 750	164/262 402	2.46 (2.10-2.88)	1.87 (1.59-2.21)	39 741	114/262 711	1.86 (1.54-2.25)	1.64 (1.35-1.98)

Abbreviations: CVD, cardiovascular disease; IRR, incidence rate ratio.

^a^ Adjusted for time since birth and calendar year.

^b^ Adjusted for time since birth, calendar year, country, maternal age at the study participant’s birth, the parents’ country of origin, highest education, and history of psychiatric disorders.

^c^ We excluded 16 865 participants from the analyses with ischemic heart disease and 16 885 from the analyses with stroke as the outcome owing to missing data on the cause of death.

^d^ The reference group was the unexposed group.

We observed some evidence for both a triggering and a long-term effect of parental death on the risk of AMI; the short-term effect was observed for parental death in adulthood (eFigure in the Supplement). There were no clear differences in the risk of stroke according to the time since loss.

### Sensitivity Analyses

Additional adjustment for the variables with large proportions of missing participants did not substantially change the studied associations (eTable 5 in the Supplement). We found no differences by country and sex in the studied associations (eTable 6 in the Supplement). The association between parental death and IHD tended to be stronger among offspring of parents with only primary education than among those with higher education. There were no such differences in cases of stroke. The results for AMI, ischemic stroke, and hemorrhagic stroke were similar to those for overall IHD and stroke, although with less statistical power (eTable 7 in the Supplement). We found no substantial changes in the associations after excluding those who lost both parents on the same day (n = 408) (eTable 8 in the Supplement) nor when restricting analyses to the first child from a family (adjusted IRRs, 1.41 [95% CI, 1.31-1.52] for IHD and 1.26 [95% CI, 1.16-1.36] for stroke).

## Discussion

In this large prospective cohort study, we found that parental death was associated with increased risks of IHD and stroke in offspring up to 43 years of age. The associations were observed not only for parental cardiovascular or other natural deaths but also for unnatural deaths. The associations were stronger in cases of the loss of both parents than the loss of 1 parent. We found no substantial differences in the studied associations according to the parent’s sex or the child’s age at loss. The risk of AMI was highest in the first 3 months after parental death in adulthood.

The finding that parental death was associated with an increased risk of IHD is in line with that of an earlier study^10^ involving a cohort of men born in 1949 to 1951 reporting an association between parental death and an increased risk of IHD up to the age of 57 to 59 years and the findings of 2 earlier studies reporting associations between parental death and the risk of CVD mortality in offspring up to 29 years of age.^17,18^ Findings from our present study also corroborate the results of several earlier investigations documenting associations between the death of other family members (ie, spouse, sibling, or child) and increased risks of AMI or IHD and stroke^3,4,6^ and of studies showing that adverse childhood experiences may be associated with increased risk of CVD later in life.^19,20^ Our study extends the limited literature on the association between parental death and the risk of severe CVD^10,17,18^ by (1) studying both sexes in a cohort born during more recent decades and thus with better societal support after parental death, (2) reducing selection bias by following study participants from birth, (3) investigating parental death both in childhood and in early adulthood, and (4) examining incident IHD and stroke, outcomes that are less prone to misclassification than cardiovascular mortality.

The findings regarding the highest risk of IHD and stroke in cases of parental death due to CVD corroborate those from an earlier study in conscripts^10^ and those of Li et al^17^ in a Nordic cohort similar to ours and are in line with the hypothesis that confounding by cardiovascular risk factors shared by family members is likely to be an important explanation for the association between bereavement and CVD; alternatively, these findings also might be indicative of increased cardiovascular risk after stress in persons with a family history of CVD as indicated by parental cardiovascular death. Nevertheless, the finding that parental unnatural deaths were also associated with increased risks of IHD and stroke may be supportive of stress-related mechanisms, as the effect of unnatural death is unlikely to be substantially confounded by familial cardiovascular risk factors.

The first few years of life are particularly sensitive to stress given the adverse effects of the prolonged separation from caregivers on the development of the brain architecture and programming of stress reactivity.^21^ During adolescence, children exposed to severe stress and limited parental supervision are vulnerable to behavioral and emotional problems^22^ and may take up adverse health behaviors related to CVD that may persist into adulthood.^23^ These hypotheses are in line with the finding that losses in childhood, particularly in the first 5 years of life and in adolescence, tended to be more strongly associated with the studied outcomes than the losses in adulthood. However, the corresponding CIs largely overlapped, a finding in line with earlier work showing no substantial differences in IHD or stroke risk according to child’s age at parental death in childhood.^10^

It is often assumed that the loss of the mother may be more detrimental than that of the father, as mothers may have stronger emotional ties with their children and are generally the primary attachment figures and main caregivers. In contrast to the earlier study involving men born from 1949 to 1951,^10^ we did not find differences in the risks of IHD and stroke according to the deceased parent’s sex, possibly owing to increased gender equality and paternal involvement in children’s upbringing during the past decades in the Nordic countries.^24^

We observed that losing a parent in childhood had a long-term impact, while losing a parent in adulthood had both short-term and long-term impacts on the risk of IHD, AMI, and stroke. Our finding of an increased risk of AMI in the first few months after parental death in adulthood is in line with the results of several studies documenting that the risk of AMI,^3^ stroke,^3^ and atrial fibrillation^2^ was higher shortly after the loss of a loved one than over the long-term. Stress is often most intense shortly after bereavement.^25^ Acute stress activates the hypothalamic-pituitary-adrenal axis and the autonomic nervous system, inducing acute changes in the neuroendocrine, inflammatory, hemostatic, and cardiovascular activity,^26,27,28^ which may increase the risk of CVD shortly after bereavement. Over the long-term, parental death, particularly in childhood, may lead to adverse health-related behaviors^12,23^ and psychosocial adversity.^8,9^ The chronic stress resulting from bereavement may lead to prolonged activation of the hypothalamic-pituitary-adrenal axis and to overactivity of the autonomic nervous system, which in turn may induce CVD.^29^

### Strengths and Limitations

Our study has several strengths. First, the registration of exposure is of high quality, with limited, if any, misclassification. The follow-up is complete, and the validity of the diagnoses is very good for AMI and good for stroke.^30,31,32^ Second, our large sample size and the detailed information on the types of exposure allowed us to contribute to a better understanding of causality by exploring dose-response patterns, sensitive periods, time periods, and groups at the highest risks of developing IHD or stroke after bereavement. Third, our follow-up from birth reduced selection bias.

Several limitations need to be noted. First, although we adjusted for several potential confounders, we cannot exclude the possibility of residual confounding. However, our findings regarding the association between parental unnatural deaths, which are less likely to be affected by cardiovascular risk factors shared by family members than those in cases of other natural deaths, and the risk of IHD and stroke are supportive of stress-related mechanisms. Second, it is not clear to what extent our findings may be generalized to other populations, eg, from other historical periods or geographical or cultural settings or to CVD in older ages. In the earlier study with individuals born in 1949 to 1951,^10^ the association between maternal unnatural deaths in childhood and the risk of incident IHD in adulthood was stronger than that observed in the present study. Similarly, in countries with no or limited support to bereaved families or limited medical resources, the association between parental death and the risk of CVD may be stronger than that in our study. Third, we did not have information on psychosocial resources, eg, the quality of relationship to the parents, the surviving parent’s coping with the loss, social support, and participation in psychotherapy or bereavement support groups,^33^ which may have buffered the detrimental association of exposure with CVD. Studies that further investigate vulnerable groups further with respect to parental bereavement are needed. Fourth, we could not study the role of psychiatric disorders in the studied association owing to lack of data on mild or untreated cases and because of uncertainties concerning the timing of presentation of the psychiatric disorders in relation to the loss.

## Conclusions

In this cohort study, death of a parent was associated with increased risks of IHD and stroke. The associations were observed not only for cardiovascular or other natural deaths but also for unnatural deaths. Family members and health professionals may need to pay attention to the cardiovascular risk among parentally bereaved children and young adults.

## Notes

Supplement. **eFigure.** Adjusted Incidence Rate Ratios and 95% Confidence Intervals for Ischemic Heart Disease, Acute Myocardial Infarction and Stroke by Time Since the Death of the Parent **eTable 1.** Registers Included in the Linkage and Retrieved Variables **eTable 2.** International Classification of Diseases Codes Used to Classify Causes of Death and Medical Conditions **eTable 3.** Adjusted Incidence Rate Ratios and 95% Confidence Intervals for Ischemic Heart Disease and Stroke According to Maternal and Paternal Death **eTable 4.** Adjusted Incidence Rate Ratios and 95% Confidence Intervals for Ischemic Heart Disease and Stroke According to the Study Participants’ Age at the Parent’s Death **eTable 5.** Adjusted Incidence Rate Ratios and 95% Confidence Intervals for Ischemic Heart Disease and Stroke by any Death of a Parent Among Participants With Data on Specific Covariates **eTable 6.** Adjusted Incidence Rate Ratios and 95% Confidence Intervals for the Association between the Death of a Parent and Ischemic Heart Disease and Stroke in Stratified Analyses **eTable 7.** Adjusted Incidence Rate Ratios and 95% Confidence Intervals for the Association between the Death of a Parent and Acute Myocardial Infarction and Ischemic and Hemorrhagic Stroke **eTable 8.** Adjusted Incidence Rate Ratios and 95% Confidence Intervals for Ischemic Heart Disease and Stroke According to Parental Death after Excluding the Participants who Lost Two Parents on the Same Day **eAppendix 1.** Description of the Source, and the Measurement of Covariates **eAppendix 2.** Description of Poisson Regression and Criteria Concerning Confounders
