# Supplementary material for: Death of a Parent and the Risk of Ischemic Heart Disease and Stroke in Denmark and Sweden
Source: JAMA Netw Open. 2022 Jun 22;5(6):e2218178. doi: 10.1001/jamanetworkopen.2022.18178 (PMC9218848; doi:10.1001/jamanetworkopen.2022.18178)
Supplement: Supplement. — eFigure. Adjusted Incidence Rate Ratios and 95% Confidence Intervals for Ischemic Heart Disease, Acute Myocardial Infarction and Stroke by Time Since the Death of the Parent eTable 1. Registers Included in the Linkage and Retrieved Variables eTable 2. International Classification of Diseases Codes Used to Classify Causes of Death and Medical Conditions eTable 3. Adjusted Incidence Rate Ratios and 95% Confidence Intervals for Ischemic Heart Disease and Stroke According to Maternal and Paternal Death eTable 4. Adjusted Incidence Rate Ratios and 95% Confidence Intervals for Ischemic Heart Disease and Stroke According to the Study Participants’ Age at the Parent’s Death eTable 5. Adjusted Incidence Rate Ratios and 95% Confidence Intervals for Ischemic Heart Disease and Stroke by any Death of a Parent Among Participants With Data on Specific Covariates eTable 6. Adjusted Incidence Rate Ratios and 95% Confidence Intervals for the Association between the Death of a Parent and Ischemic Heart Disease and Stroke in Stratified Analyses eTable 7. Adjusted Incidence Rate Ratios and 95% Confidence Intervals for the Association between the Death of a Parent and Acute Myocardial Infarction and Ischemic and Hemorrhagic Stroke eTable 8. Adjusted Incidence Rate Ratios and 95% Confidence Intervals for Ischemic Heart Disease and Stroke According to Parental Death after Excluding the Participants who Lost Two Parents on the Same Day eAppendix 1. Description of the Source, and the Measurement of Covariates eAppendix 2. Description of Poisson Regression and Criteria Concerning Confounders [file jamanetwopen-e2218178-s001.pdf]

# Supplemental Online Content

Chen H, Li J, Wei D, et al. Death of a parent and the risk of ischemic heart disease and stroke in Denmark and Sweden. *JAMA Netw Open*. 2022;5(6):e2218178. doi:10.1001/jamanetworkopen.2022.18178

**eFigure.** Adjusted Incidence Rate Ratios and 95% Confidence Intervals for Ischemic Heart Disease, Acute Myocardial Infarction and Stroke by Time Since the Death of the Parent

**eTable 1.** Registers Included in the Linkage and Retrieved Variables

**eTable 2.** International Classification of Diseases Codes Used to Classify Causes of Death and Medical Conditions

**eTable 3.** Adjusted Incidence Rate Ratios and 95% Confidence Intervals for Ischemic Heart Disease and Stroke According to Maternal and Paternal Death

**eTable 4.** Adjusted Incidence Rate Ratios and 95% Confidence Intervals for Ischemic Heart Disease and Stroke According to the Study Participants' Age at the Parent's Death

**eTable 5.** Adjusted Incidence Rate Ratios and 95% Confidence Intervals for Ischemic Heart Disease and Stroke by any Death of a Parent Among Participants With Data on Specific Covariates

**eTable 6.** Adjusted Incidence Rate Ratios and 95% Confidence Intervals for the Association between the Death of a Parent and Ischemic Heart Disease and Stroke in Stratified Analyses

**eTable 7.** Adjusted Incidence Rate Ratios and 95% Confidence Intervals for the Association between the Death of a Parent and Acute Myocardial Infarction and Ischemic and Hemorrhagic Stroke

**eTable 8.** Adjusted Incidence Rate Ratios and 95% Confidence Intervals for Ischemic Heart Disease and Stroke According to Parental Death after Excluding the Participants who Lost Two Parents on the Same Day

**eAppendix 1.** Description of the Source, and the Measurement of Covariates

**eAppendix 2.** Description of Poisson Regression and Criteria Concerning Confounders

This supplemental material has been provided by the authors to give readers additional information about their work.

© 2022 Chen H et al. *JAMA Network Open.*

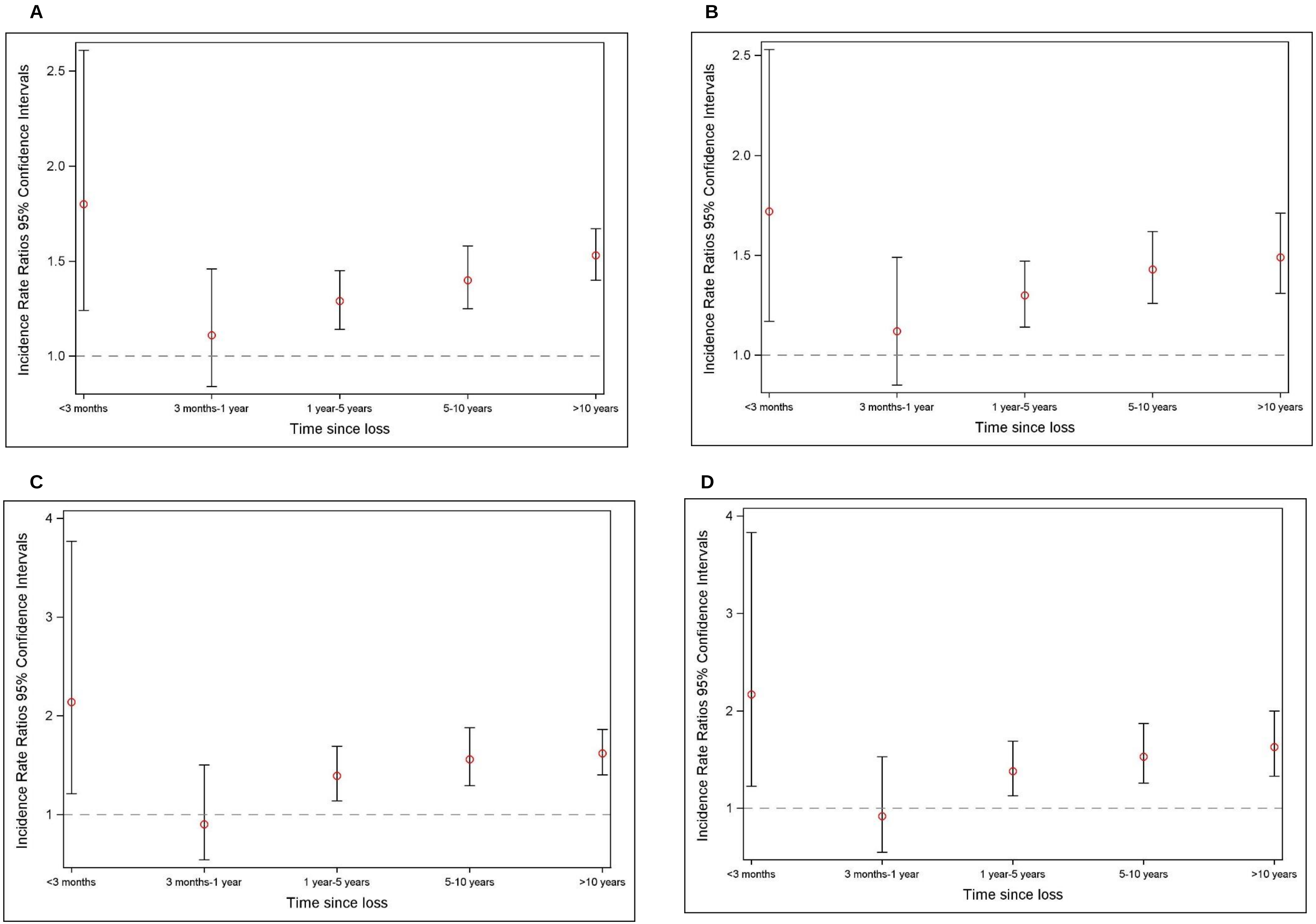


© 2022 Chen H et al. *JAMA Network Open.*

E

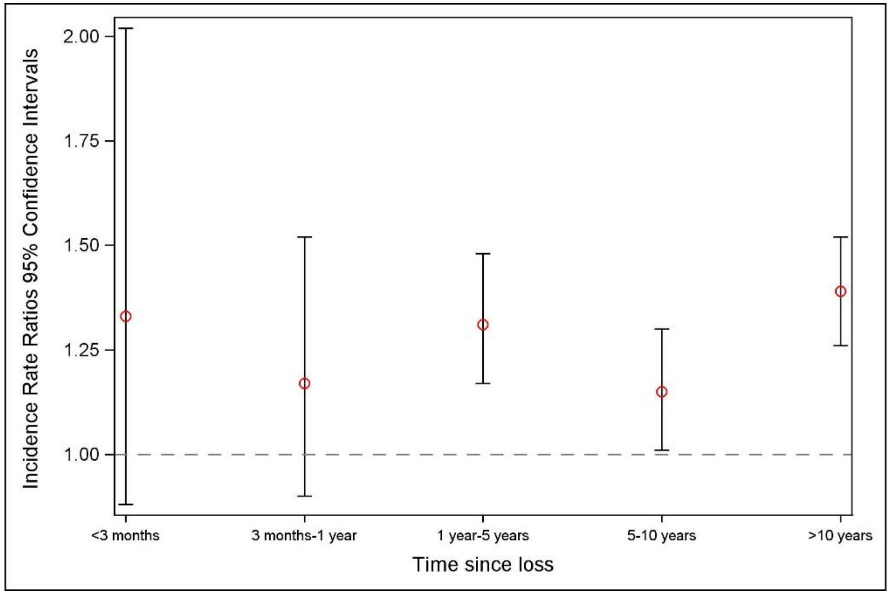


F

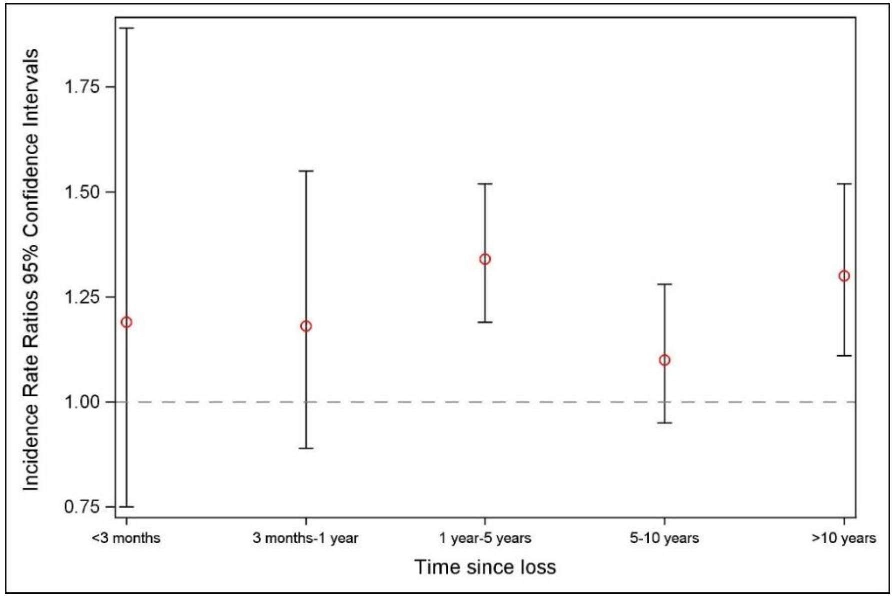


**eFigure. Adjusted Incidence Rate Ratios and 95% Confidence Intervals for Ischemic Heart Disease, Acute Myocardial Infarction and Stroke by Time Since the Death of the Parent** (A) Any loss and ischemic heart disease; (B) Any loss after the age of 18 and ischemic heart disease; (C) Any loss and acute myocardial infarction; (D) Any loss after the age of 18 and acute myocardial infarction; (E) Any loss and stroke; (F) Any loss after the age of 18 and stroke. We adjusted for time since birth, calendar year, country, maternal age at the study participants' birth, the parents' country of origin, highest education and history of psychiatric disorders; the reference group was the unexposed.

© 2022 Chen H et al. *JAMA Network Open.*

## eTable 1. Registers Included in the Linkage and Retrieved Variables

| Registers | Retrieved variables | Period with available data |
|---|---|---|
| Denmark | | |
| Medical Birth Register | Sex | 1973-2016 |
| | The study participants' date of birth | 1973–2016 |
| | Gestational age | 1978-2016 |
| | Linkage to mother | 1973-2016 |
| | Linkage to the father | 1991-2016 |
| | Maternal age at the study participants' birth | 1973-2016 |
| | Maternal smoking | 1991-2016 |
| Civil Registration System | Sex | 1973-2016 |
| | The study participants' date of birth | 1973-2016 |
| | The mother's date of birth | 1968-2016 |
| | Parents' country of origin | 1986-2016 |
| | Date and cause of death | 1970-2016 |
| | Linkage to parents, grandparents and parents' siblings | 1968-2016 |
| | Emigration date | 1973-2016 |
| National Hospital Register[a] | Date and diagnosis of hospitalization/specialized outpatient care | 1977-2016 |
| Psychiatric Central Register[b] | Date and diagnosis of psychiatric hospitalizations/outpatient psychiatric care | 1969-2016 |
| The Integrated Database for Longitudinal Labor Market Research | Education | 1980-2016 |
| | Income | 1980-2015 |
| Sweden | | |
| Medical Birth Register | Sex | 1973-2014 |
| | The study participants' date of birth | 1973-2014 |
| | Gestational age | 1973-2014 |
| | Linkage to the mother | 1973-2014 |
| | Maternal smoking | 1982-2014 |
| | Maternal height in early pregnancy | 1982-2014 |
| | Maternal weight in early pregnancy | 1982-1989, 1992-2014 |
| | Maternal hypertension and diabetes before or during pregnancy | 1973-2014 |
| Total Population Register | The mother's year of birth | 1973-2014 |
| | The parents' country of origin | 1973-2014 |
| | Emigration date | 1973-2014 |
| Multi-Generation Register | Linkages to parents, grandparents and parents' siblings | 1961-2014 |
| Cause of Death Register | Date and cause of death | 1952-2014 |
| Patient Register[c] | Date and diagnosis of hospitalization/ Specialized outpatient care | 1969-2014 |
| Education Register | Education | 1990-2014 |
| Register of Incomes and Taxes | Personal income | 1972-2014 |

[a]Includes all inpatient diagnoses since 1977 and all specialized outpatient diagnoses since 1995.
[b]Includes data on psychiatric hospitalizations since 1969 and on outpatient psychiatric care since 1995.
[c]Inpatient diagnoses are available since 1969, with the coverage of the inpatient care becoming nationwide in 1987; data on specialized outpatient care are included from 2001.

© 2022 Chen H et al. *JAMA Network Open.*

## eTable 2. International Classification of Diseases Codes Used to Classify Causes of Death and Medical Conditions

| Medical condition | ICD-codes | | | | |
|---|---|---|---|---|---|
| | ICD-6[a] | ICD-7[a] | ICD-8[a] | ICD-9[a] | ICD-10[a] |
| Denmark | | | | | |
| Cause of death of the parent | | | | | |
| Cardiovascular death | - | - | 390-458 | - | I00-I99 |
| Other natural death | - | - | The rest of the codes | - | The rest of the codes |
| Unnatural death | - | - | 7959, 79621, 800-999 | - | R95, R96, R98, V01-Y98 |
| The outcomes of interest | | | | | |
| Ischemic heart disease | - | - | 410-414 | - | I20-I25 |
| Acute myocardial infarction | | | 410 | - | I21, I22 |
| Stroke | - | - | 430, 431, 433, 434, 436 | - | I60, I61, I63, I64 |
| Ischemic stroke | | | 433, 434 | - | I63 |
| Hemorrhagic stroke | | | | | |
| Subarachnoid hemorrhage | - | - | 430 | - | I60 |
| Intracerebral hemorrhage | - | - | 431 | - | I61 |
| Maternal hypertension | - | - | 400-404, 63700, 63703, 63704, 63709, 63719 | - | I10-I15, O10, O11, O13-O16 |
| Maternal diabetes | - | - | 249, 250 | - | E10-E14, O24 |
| Cardiovascular diseases of parents | - | - | 390-458 | - | I00-I99 |
| Psychiatric diseases of parents | - | - | 290-315 | - | F00-F99 |
| Cardiovascular diseases in the family | - | - | 390-458 | - | I00-I99 |
| Psychiatric diseases in the family | - | - | 290-315 | - | F00-F99 |
| Sweden | | | | | |
| Cause of death of the parent | - | - | | | |
| Cardiovascular death | - | - | 390-458 | 390-459 | I00-I99 |
| Other natural death | - | - | The rest of the codes | The rest of the codes | The rest of the codes |
| Unnatural death | - | - | 7959, 79621 , 800-999 | 798, 800-999 | R95, R96, R98, V01-Y98 |
| The outcomes of interest | | | | | |
| Ischemic heart disease | - | - | 410-414 | 410-414 | I20-I25 |
| Acute myocardial infarction | - | - | 410 | 410 | I21, I22 |
| Stroke | - | - | 430, 431, 433, 434, 436 | 430, 431, 434, 436 | I60, I61, I63, I64 |
| Ischemic stroke | - | - | 433, 434 | 434 | I63 |
| Hemorrhagic stroke | | | | | |
| Subarachnoid hemorrhage | - | - | 430 | 430 | I60 |
| Intracerebral hemorrhage | - | - | 431 | 431 | I61 |
| Maternal hypertension | - | - | 400-404, 63701, 63703, | 401-405, 642 | - |

© 2022 Chen H et al. *JAMA Network Open.*

| | | | | | |
|---|---|---|---|---|---|
| | | | 63704, 63709, 63710 | | |
| Maternal diabetes | - | - | 250 | 250, 648A | - |
| Cardiovascular diseases of parents | - | - | 390-458 | 390-459 | - |
| Psychiatric diseases of parents | - | - | 290-315 | 290-319 | - |
| Cardiovascular diseases in family | 400-468 | 400-468 | 390-458 | 390-459 | - |
| Psychiatric diseases in family | - | - | 290-315 | 290-319 | - |

ICD=International Classification of Diseases.
[a]In Denmark ICD-8 was used during 1970-1993, while ICD-10 during 1994-2016. In Sweden ICD-6 was used during 1952-1957, ICD-7 during 1958-1968, ICD-8 during 1969-1986, ICD-9 during 1987-1996 9 and ICD-10 during 1997-2014.

© 2022 Chen H et al. *JAMA Network Open.*

**eTable 3. Adjusted Incidence Rate Ratios and 95% Confidence Intervals for Ischemic Heart Disease and Stroke According to Maternal and Paternal Death**

| Exposure | Ischemic heart disease | | | Stroke | | |
|---|---|---|---|---|---|---|
| | N | Events /person-years | Adjusted IRR (95% CI)[a] | N | Events / person-years | Adjusted IRR (95% CI)[a] |
| Unexposed | 3 286 040 | 4,609 /103 286 808 | 1.00 | 3 286 224 | 7002/ 103 256 190 | 1.00 |
| Exposed to mother's death | | | | | | |
| Cause of death[b,c] | | | | | | |
| Death due to CVD | 19 771 | 96/206 574 | 2.46 (2.01-3.02) | 19 752 | 60/206 660 | 1.58 (1.22-2.05) |
| Other natural death | 110 328 | 257/1 139 153 | 1.18 (1.04-1.34) | 110 286 | 264/1 138 676 | 1.28 (1.12-1.45) |
| Unnatural death | 18 210 | 43/275 893 | 1.17 (0.86-1.58) | 18 207 | 54/275 763 | 1.41 (1.08-1.85) |
| Participants' age at loss (in years)[c] | | | | | | |
| 0-5 | 7 557 | 23/193 446 | 1.59 (1.04-2.42) | 7 557 | 17/193 493 | 1.04 (0.65-1.68) |
| 6-12 | 16 847 | 36/341 761 | 1.06 (0.76-1.49) | 16 847 | 52/341 610 | 1.37 (1.03-1.81) |
| 13-18 | 24 190 | 66/344 484 | 1.50 (1.18-1.92) | 24 187 | 64/344 400 | 1.31 (1.02-1.68) |
| 19-25 | 37 124 | 94/385 433 | 1.28 (1.04-1.57) | 37 110 | 113/385 146 | 1.52 (1.26-1.84) |
| 26-30 | 26 784 | 89/202 647 | 1.52 (1.23-1.88) | 26 758 | 69/202 550 | 1.33 (1.05-1.70) |
| >30 | 41 042 | 94/160 878 | 1.26 (1.02-1.55) | 41 037 | 66/160 924 | 1.17 (0.91-1.50) |
| Exposed to father's death | | | | | | |
| Cause of death[b,c] | | | | | | |
| Death due to CVD | 79 187 | 326/855 594 | 2.22 (1.98-2.49) | 79 160 | 217/855 693 | 1.47 (1.28-1.69) |
| Other natural death | 180 412 | 425/1 789 072 | 1.18 (1.07-1.31) | 180 304 | 390/1 788 289 | 1.17 (1.05-1.30) |
| Unnatural death | 56 105 | 140/910 281 | 1.29 (1.09-1.53) | 56 100 | 160/909 931 | 1.36 (1.16-1.60) |
| Participants' age at loss (in years)[c] | | | | | | |
| 0-5 | 20 385 | 62/540 519 | 1.56 (1.20-2.03) | 20 385 | 68/540 330 | 1.48 (1.16-1.88) |
| 6-12 | 37 367 | 106/761 751 | 1.48 (1.22-1.80) | 37 361 | 109/761 583 | 1.33 (1.10-1.61) |
| 13-18 | 51 821 | 153/737 750 | 1.62 (1.38-1.90) | 51 801 | 155/737 385 | 1.48 (1.26-1.73) |
| 19-25 | 77 601 | 207/791 367 | 1.37 (1.19-1.58) | 77 561 | 168/791 085 | 1.11 (0.95-1.30) |
| 26-30 | 55 711 | 186/417 494 | 1.56 (1.34-1.81) | 55 681 | 120/417 515 | 1.13 (0.94-1.36) |
| >30 | 84 449 | 190/329 718 | 1.26 (1.09-1.47) | 84 409 | 151/329 641 | 1.32 (1.11-1.56) |

IRR=incidence rate ratio; CI=confidence intervals; CVD=cardiovascular diseases.
[a]Adjusted for time since birth, calendar year, country, maternal age at the study participants' birth, the parents' country of origin, highest education and history of psychiatric disorders.
[b] We excluded 16 865 participants from the analyses with ischemic heart disease and 16 885 from the analyses with stroke as the outcome, due to missing data on the cause of death.
[c]The reference group was the unexposed.

© 2022 Chen H et al. *JAMA Network Open.*

**eTable 4. Adjusted Incidence Rate Ratios and 95% Confidence Intervals for Ischemic Heart Disease and Stroke According to the Study Participants' Age at the Parent's Death**

| Exposure | Ischemic heart disease | | | | Stroke | |
|---|---|---|---|---|---|---|
| | **N** | **Events /person-years** | **Adjusted IRR (95% CI)[a]** | **N** | **Events /person-years** | **Adjusted IRR (95% CI)[a]** |
| Unexposed | 3 286 040 | 4609 /103 286 808 | 1.00 | 3 286 224 | 7002/ 103 256 190 | 1.00 |
| Participants' age at loss | | | | | | |
| ≤18 years[b] | 158167 | 446/2 919 711 | 1.50 (1.36-1.66) | 158 138 | 465/2 918 801 | 1.38 (1.26-1.52) |
| Cause of death[b,c] | | | | | | |
| Death due to CVD | 29 289 | 152/535 616 | 2.75 (2.33-3.25) | 29 279 | 107/535 645 | 1.70 (1.40-2.06) |
| Other natural death | 80 913 | 174/1 425 538 | 1.17 (1.00-1.36) | 80 901 | 201/1 425 040 | 1.19 (1.03-1.37) |
| Unnatural death | 46 837 | 119/948 923 | 1.30 (1.08-1.57) | 46 831 | 156/948 506 | 1.51 (1.28-1.77) |
| Sex of the deceased parent[b] | | | | | | |
| Mother | 48 594 | 125/879 692 | 1.36 (1.14-1.63) | 48 591 | 133/879 502 | 1.29 (1.08-1.53) |
| Father | 109 573 | 321/2 040 020 | 1.56 (1.39-1.76) | 109 547 | 332/2 039 299 | 1.43 (1.28-1.60) |
| >18 years[b] | 322 711 | 860/2 287 537 | 1.38 (1.28-1.49) | 322 556 | 687/2 286 861 | 1.25 (1.15-1.36) |
| Cause of death[b,c] | | | | | | |
| Death due to CVD | 69 669 | 270/526 551 | 2.08 (1.83-2.35) | 69 633 | 170/526 708 | 1.40 (1.20-1.63) |
| Other natural death | 209 827 | 508/1 502 688 | 1.19 (1.08-1.31) | 209 689 | 453/1 501 925 | 1.22 (1.11-1.35) |
| Unnatural death | 27 478 | 64/237 251 | 1.18 (0.92-1.51) | 27 476 | 58/237 188 | 1.12 (0.87-1.45) |
| Sex of the deceased parent[b] | | | | | | |
| Mother | 104 950 | 277/748 957 | 1.35 (1.19-1.53) | 104 905 | 248/748 620 | 1.37 (1.20-1.56) |
| Father | 217 761 | 583/1 538 579 | 1.40 (1.28-1.53) | 217 651 | 439/1 538 241 | 1.19 (1.07-1.31) |

IRR=incidence rate ratio; CI=confidence intervals; CVD=cardiovascular diseases.
[a]Adjusted for time since birth, calendar year, country, maternal age at the study participants' birth and the parents' country of origin, highest education and history of psychiatric disorders.
[b]The reference group was the unexposed.
[c]We excluded 16 865 participants from the analyses with ischemic heart disease and 16 885 from the analyses with stroke as the outcome, due to missing data on the cause of death

© 2022 Chen H et al. *JAMA Network Open.*

## eTable 5. Adjusted Incidence Rate Ratios and 95% Confidence Intervals for Ischemic Heart Disease and Stroke by Any Death of a Parent Among Participants With Data on Specific Covariates

| Covariates additionally adjusted for | N1[a] | | N2[b] | Ischemic heart disease | | Stroke | |
|---|---|---|---|---|---|---|---|
| | Without missing | Missing | | Main model[c] | Main model[c]+ covariate | Main model[c] | Main model[c]+ covariate |
| | | | | IRR (95% CI) | IRR (95% CI) | IRR (95% CI) | IRR (95% CI) |
| The study participants' gestational age at birth | 3 357 600 | 409 318 | 3 347 485 | 1.39 (1.26-1.54) | 1.39 (1.25-1.53) | 1.32 (1.21-1.44) | 1.31 (1.21-1.43) |
| Maternal income at the study participants' birth | 3 622 476 | 144 442 | 3 168 547 | 1.40 (1.29-1.51) | 1.39 (1.29-1.50) | 1.31 (1.22-1.41) | 1.31 (1.22-1.41) |
| Maternal smoking in early pregnancy | 1 719 488 | 2 047 430 | 1 716 510 | 1.46 (1.04-2.07) | 1.44 (1.02-2.04) | 1.14 (0.90-1.44) | 1.11 (0.87-1.41) |
| Maternal BMI in early pregnancy | 840 993 | 2 925 925 | 840 246 | 1.20 (0.66-2.18) | 1.20 (0.66-2.17) | 1.16 (0.83-1.61) | 1.16 (0.83-1.61) |
| Maternal hypertension before or during pregnancy | 3 488 704 | 278 214 | 3 477 735 | 1.41 (1.29-1.54) | 1.41 (1.29-1.54) | 1.30 (1.20-1.41) | 1.30 (1.20-1.41) |
| Maternal diabetes before or during pregnancy | 3 488 704 | 278 214 | 3 477 735 | 1.41 (1.29-1.54) | 1.41 (1.29-1.54) | 1.30 (1.20-1.41) | 1.30 (1.20-1.41) |
| Parents' CVD before the study participants' birth | 3 488 704 | 278 214 | 3 477 735 | 1.41 (1.29-1.54) | 1.41 (1.29-1.54) | 1.30 (1.20-1.41) | 1.30 (1.20-1.41) |
| Family history of CVD before the study participants' birth | 2 706 528 | 1 060 390 | 2 704 822 | 1.42 (1.24-1.63) | 1.41 (1.23-1.61) | 1.27 (1.14-1.43) | 1.27 (1.14-1.42) |
| Family history of psychiatric disorders before the study participants' birth | 2 602 006 | 1 164 912 | 2 600 565 | 1.44 (1.25-1.66) | 1.43 (1.23-1.65) | 1.28 (1.14-1.44) | 1.28 (1.14-1.44) |

IRR=incidence rate ratio; CI=confidence intervals; BMI =body-mass index; CVD=cardiovascular diseases.
[a]The number of participants without and with missing data for each covariate additionally adjusted for.
[b]The number of participants without missing data on each covariate in the fully adjusted model.
[c]Adjusted for time since birth, calendar year, country, maternal age at the study participants' birth, the parents' country of origin, highest education and history of psychiatric disorders.

© 2022 Chen H et al. *JAMA Network Open.*

**eTable 6. Adjusted Incidence Rate Ratios and 95% Confidence Intervals for the Association between the Death of a Parent and Ischemic Heart Disease and Stroke in Stratified Analyses**

| Stratification variable | Ischemic heart disease | | Stroke | |
|---|---|---|---|---|
| | Events/person-years | Adjusted IRR (95% CI)[a] | Events/person-years | Adjusted IRR (95% CI)[a] |
| Country | | | | |
| Denmark | | | | |
| Unexposed | 3570/43 064 363 | 1.00 | 3994/43 052 698 | 1.00 |
| Any death | 1091/2 600 129 | 1.40 (1.31-1.51) | 786/2 600 329 | 1.29 (1.19-1.40) |
| Sweden | | | | |
| Unexposed | 1039/60 222 445 | 1.00 | 3008/60 203 492 | 1.00 |
| Any death | 215/2 607 119 | 1.46 (1.25-1.71) | 366/2 605 333 | 1.29 (1.15-1.44) |
| Highest parental education | | | | |
| 0-9 years | | | | |
| Unexposed | 827/9 687 675 | 1.00 | 960/9 684 997 | 1.00 |
| Any death | 457/1 111 884 | 1.61 (1.42-1.82) | 328/1 112 272 | 1.31 (1.14-1.49) |
| 10-14 years | | | | |
| Unexposed | 2758/62 992 500 | 1.00 | 4284/62 973 730 | 1.00 |
| Any death | 652/2 993 498 | 1.33 (1.22-1.45) | 618/2 992 179 | 1.25 (1.15-1.37) |
| ≥15 years | | | | |
| Unexposed | 1019/30 497 247 | 1.00 | 1752/30 488 112 | 1.00 |
| Any death | 190/1 077 560 | 1.39 (1.18-1.63) | 203/1 076 873 | 1.39 (1.20-1.62) |
| Sex of the child | | | | |
| Men | | | | |
| Unexposed | 2990/52 992 588 | 1.00 | 3625/52 980 451 | 1.00 |
| Any death | 829/2 682 096 | 1.37 (1.27-1.49) | 577/2 682 765 | 1.25 (1.13-1.37) |
| Mother's death | 242/843 051 | 1.24 (1.08 -1.42) | 174/843 303 | 1.17 (1.00-1.37) |
| Father's death | 587/1 839 045 | 1.44 (1.31-1.58) | 403/1 839 462 | 1.28 (1.15-1.43) |
| Women | | | | |
| Unexposed | 1619/50 292 051 | 1.00 | 3377/50 273 570 | 1.00 |
| Any death | 477/2 525 071 | 1.49 (1.34-1.67) | 575/2 522 815 | 1.35 (1.23-1.48) |
| Mother's death | 160/785 573 | 1.55 (1.31-1.84) | 207/784 793 | 1.52 (1.32-1.76) |
| Father' death | 317/1 739 498 | 1.46 (1.29-1.66) | 368/1 738 022 | 1.27 (1.14-1.42) |

IRR=incidence rate ratio; CI=confidence intervals; CVD=cardiovascular diseases.
[a]Adjusted for time since birth, calendar year, maternal age at the study participants' birth, country, the parents' country of origin, highest education and history of psychiatric disorders.

© 2022 Chen H et al. *JAMA Network Open.*

**eTable 7. Adjusted Incidence Rate Ratios and 95% Confidence Intervals for the Association between the Death of a Parent and Acute Myocardial Infarction and Ischemic and Hemorrhagic Stroke**

| Exposure | Acute myocardial infarction | | Ischemic stroke | | Hemorrhagic stroke | | | |
|---|---|---|---|---|---|---|---|---|
| | | | | | Subarachnoid hemorrhage | | Intracerebral hemorrhage | |
| | Events/person-years | Adjusted IRR (95% CI)[a] | Events/person-years | Adjusted IRR (95% CI)[a] | Events/person-years | Adjusted IRR (95% CI)[a] | Events/person-years | Adjusted IRR (95% CI)[a] |
| Unexposed | 1507 /103 306 969 | 1.00 | 2741 /103 294 810 | 1.00 | 1940/ 103 296 764 | 1.00 | 1798/ 103 297 769 | 1.00 |
| Any death | 510/5 213 021 | 1.51 (1.36-1.68) | 537/5 212 060 | 1.32 (1.20-1.46) | 261/5 212 700 | 1.25 (1.09-1.44) | 206/5 213 215 | 1.17 (1.01-1.36) |
| Cause of death[b,c] | | | | | | | | |
| Death due to CVD | 182/1 063 837 | 2.57 (2.19-3.02) | 143/1 063 641 | 1.65 (1.39-1.96) | 53/1 064 096 | 1.22 (0.92-1.61) | 54/1 064 143 | 1.47 (1.12-1.94) |
| Other natural death | 259/2 931 397 | 1.22 (1.07-1.40) | 303/2 930 744 | 1.21 (1.07-1.37) | 142/2 931 088 | 1.15 (0.96-1.37) | 106/2 931 408 | 1.01 (0.82-1.24) |
| Unnatural death | 62/1 187 029 | 1.25 (0.96-1.61) | 87/1 186 914 | 1.30 (1.05-1.62) | 64/1 186 783 | 1.61 (1.25-2.07) | 45/1 186 909 | 1.37 (1.02-1.85) |
| Participants' age at loss (in years)[c] | | | | | | | | |
| 0-5 | 30/734 377 | 1.80 (1.23-2.64) | 37/734 331 | 1.52 (1.10-2.11) | 25/734 320 | 1.41 (0.94-2.10) | 15/734 383 | 1.02 (0.61-1.71) |
| 6-12 | 48/1 104 224 | 1.42 (1.05-1.90) | 68/1 104 029 | 1.44 (1.13-1.83) | 41/1 104 074 | 1.20 (0.87-1.65) | 34/1 104 102 | 1.27 (0.90-1.79) |
| 13-18 | 79/1 083 077 | 1.67 (1.33-2.10) | 94/1 082 937 | 1.47 (1.19-1.80) | 55/1 082 981 | 1.39 (1.06-1.82) | 42/1 083 026 | 1.28 (0.94-1.74) |
| 19-25 | 107/1 178 163 | 1.34 (1.10-1.63) | 124/1 177 843 | 1.23 (1.02-1.47) | 63/1 178 029 | 1.18 (0.92-1.53) | 42/1 178 189 | 0.95 (0.70-1.30) |
| 26-30 | 119/621 247 | 1.76 (1.45-2.13) | 91/621 150 | 1.17 (0.95-1.45) | 40/621 260 | 1.23 (0.89-1.70) | 33/621 316 | 1.16 (0.81-1.64) |
| >30 | 127/491 932 | 1.38 (1.14-1.67) | 123/491 770 | 1.32 (1.10-1.60) | 37/492 035 | 1.17 (0.83-1.64) | 40/492 199 | 1.41 (1.01-1.96) |
| Sex of the deceased parentc | | | | | | | | |
| Mother | 154/1 630 564 | 1.42 (1.20-1.68) | 178/1 630 218 | 1.37 (1.17-1.60) | 81/1 630 525 | 1.20 (0.95-1.51) | 69/1 630 635 | 1.24 (0.97-1.58) |
| Father | 356/3 582 458 | 1.56 (1.38-1.76) | 359/3 581 842 | 1.30 (1.16-1.45) | 180/3 582 174 | 1.28 (1.09-1.50) | 137/3 582 580 | 1.14 (0.95-1.37) |

IRR=incidence rate ratio; CI=confidence intervals; CVD=cardiovascular diseases.

[a]Adjusted for time since birth, calendar year, maternal age at the study participants' birth, country, the parents' country of origin, highest education and history of psychiatric disorders.[b]We excluded 16 930 participants from the analyses with acute myocardial infarction, 16 937 from the analyses with ischemic stroke, 16 929 from the analyses with subarachnoid hemorrhage and 16 938 from the analyses with intracerebral hemorrhage as the outcome, due to missing data on the cause of death.

[c]The reference group was the unexposed.

© 2022 Chen H et al. *JAMA Network Open.*

**eTable 8. Adjusted Incidence Rate Ratios and 95% Confidence Intervals for Ischemic Heart Disease and Stroke According to Parental Death after Excluding the Participants Who Lost Two Parents on the Same Day**

| **Exposure** | | **Ischemic heart disease** | | | **Stroke** | |
|---|---|---|---|---|---|---|
| | **N** | **Events/ person-years** | **Adjusted IRR (95% CI)[a]** | **N** | **Events / person-years** | **Adjusted IRR (95% CI)[a]** |
| Unexposed | 3 286 040 | 4609/103 280 653 | 1.00 | 3 286 224 | 7002/103 250 034 | 1.00 |
| All deaths | 480 470 | 1305/5 203 023 | 1.41 (1.32-1.51) | 480 286 | 1152/5 201 427 | 1.30 (1.21-1.39) |
| Cause of death[b,c] | | | | | | |
| Death due to CVD | 98 951 | 422/1 062 106 | 2.27 (2.05-2.52) | 98 905 | 277/1 062 291 | 1.50 (1.32-1.69) |
| Other natural death | 290 707 | 682/2 927 973 | 1.18 (1.09-1.28) | 290 557 | 654/2 926 713 | 1.21 (1.11-1.32) |
| Unnatural death | 73 977 | 183/1 182 459 | 1.26 (1.09-1.46) | 73 969 | 214/1 181 979 | 1.38 (1.20-1.58) |
| Participants' age at loss (in years)[c] | | | | | | |
| 0-5 | 27 856 | 85/732 944 | 1.57 (1.25-1.97) | 27 856 | 85/732 802 | 1.36 (1.10-1.69) |
| 6-12 | 54 108 | 142/1 102 218 | 1.35 (1.14-1.61) | 54 102 | 161/1 101 898 | 1.34 (1.14-1.57) |
| 13-18 | 75 941 | 218/1 081 509 | 1.58 (1.37-1.81) | 75 918 | 219/1 081 052 | 1.43 (1.24-1.63) |
| 19-25 | 114 645 | 301/1 176 087 | 1.34 (1.19-1.51) | 114 591 | 281/1 175 518 | 1.25 (1.10-1.41) |
| 26-30 | 82 456 | 275/619 805 | 1.55 (1.36-1.75) | 82 400 | 189/619 730 | 1.20 (1.04-1.39) |
| >30 | 125 464 | 284/490 459 | 1.26 (1.11-1.43) | 125 419 | 217/490 427 | 1.27 (1.10-1.46) |
| Sex of the deceased parent[c] | | | | | | |
| Mother | 153 136 | 401/1 624 423 | 1.35 (1.21-1.50) | 153 088 | 381/1 623 888 | 1.34 (1.20-1.49) |
| Father | 327 334 | 904/3 578 599 | 1.45 (1.34-1.56) | 327 198 | 771/3 577 540 | 1.28 (1.18-1.38) |
| Number of deceased parents during the study period[c] | | | | | | |
| One parent | 441 128 | 1142/4 944 847 | 1.37 (1.28-1.47) | 440 953 | 1038/4 942 951 | 1.27 (1.19-1.36) |
| Both parents | 39 342 | 163/258 176 | 1.87 (1.59-2.20) | 39 333 | 114/258 476 | 1.65 (1.36-2.00) |

IRR=incidence rate ratio; CI=confidence intervals; CVD=cardiovascular diseases.
[a]Adjusted for time since birth, calendar year, country, maternal age at the study participants' birth, the parents' country of origin, highest education and history of psychiatric disorders.
[b]We excluded 16 835 participants from the analyses with ischemic heart disease and 16 855 from the analyses with stroke as the outcome, due to missing data on the cause of death.
[c]The reference group was the unexposed.

© 2022 Chen H et al. *JAMA Network Open.*

## eAppendix 1. Description of the Source, and the Measurement of Covariates

We retrieved information on participants' sex and date of birth, maternal age at participants' birth and parents' country of origin from the Danish Civil Registration System, the DMBR, the Swedish Total Population Register and the SMBR. We obtained information on parents' highest education and maternal income from the Integrated Database for Labor Market Research in Denmark and from the Education Register and the Register of Incomes and Taxes, respectively in Sweden. We defined parental education as the highest education of the two parents; if one of the parents had missing information on education, we used information on education from the other parent. We defined maternal income based on information from the year of the study participants' birth; if information for this year was missing, we used information from the closest year with available data in the five years before or after birth year; we gave priority to the income after birth year. We retrieved information on maternal smoking and gestational age at birth from MBRs in both countries and height and weight in early pregnancy from the SMBR. We calculated maternal body-mass index (BMI) by dividing weight (in kilograms) by the square of height (in meters). We obtained information on maternal hypertension and diabetes before or during pregnancy and on parents' history of CVD from the National Hospital Register in Denmark and from the SMBR and the Patient Register, respectively in Sweden. We obtained information on family (grandparents and uncles/aunts) history of CVD from the National Hospital Register and the Civil Registration System in Denmark and the Patient Register and the Cause of Death Register in Sweden. We obtained information on parental and family history of psychiatric disorders from the National Hospital Register and the Psychiatric Central Register in Denmark and from the Patient Register in Sweden.

## eAppendix 2. Description of Poisson Regression and Criteria Concerning Confounders

We treated exposure, time since birth and calendar year of follow-up as time-varying variables in our multivariable Poisson models. Study participants who lost a parent contributed person-time from birth until the death of a parent to the unexposed group and to the exposed group afterwards; unexposed participants contributed person-time to the unexposed group. We split follow-up by time since birth (at every 5 years), calendar year (categorized as 1973-1979, and 10-year intervals from 1980-2016) and exposure, leading to each study participant having multiple rows with observations in the dataset. Follow-up time was calculated from the date of entry to the date of exit in each of the resulting time spans; Poisson regression considers the clustering of multiple time spans for each study participants. We set the logarithm of the follow-up time as the offset in the Poisson regression models.

We chose a variable as a confounder in the multivariable model if there was (1) a known or an a-priori considered plausible association with the death of a parent and the risk of ischemic heart diseases and stroke and (2) it was not on the pathway between exposure and the outcomes.

© 2022 Chen H et al. *JAMA Network Open.*
